# Improving structure-based protein-ligand affinity prediction by graph representation learning and ensemble learning

**DOI:** 10.1371/journal.pone.0296676

**Published:** 2024-01-17

**Authors:** Jia Guo

**Affiliations:** 1 Chongqing Institute of Green and Intelligent Technology, Chinese Academy of Sciences, Beijing, P.R. China; 2 Chongqing School, University of Chinese Academy of Sciences, Chongqing, China; The Islamia University of Bahawalpur Pakistan, PAKISTAN

## Abstract

Predicting protein-ligand binding affinity presents a viable solution for accelerating the discovery of new lead compounds. The recent widespread application of machine learning approaches, especially graph neural networks, has brought new advancements in this field. However, some existing structure-based methods treat protein macromolecules and ligand small molecules in the same way and ignore the data heterogeneity, potentially leading to incomplete exploration of the biochemical information of ligands. In this work, we propose LGN, a graph neural network-based fusion model with extra ligand feature extraction to effectively capture local features and global features within the protein-ligand complex, and make use of interaction fingerprints. By combining the ligand-based features and interaction fingerprints, LGN achieves Pearson correlation coefficients of up to 0.842 on the PDBbind 2016 core set, compared to 0.807 when using the features of complex graphs alone. Finally, we verify the rationalization and generalization of our model through comprehensive experiments. We also compare our model with state-of-the-art baseline methods, which validates the superiority of our model. To reduce the impact of data similarity, we increase the robustness of the model by incorporating ensemble learning.

## Introduction

The prediction of binding affinity between proteins and ligands has been a longstanding area of interest in the field of virtual drug discovery. Modeling of protein-ligand interaction can be seen as a molecular docking task, a theoretical simulation method that focuses on how molecules (e.g., proteins and ligands) interact with each other [1]. Methods for measuring the protein-ligand binding can be collectively referred to as scoring function [2]. Its primary applications encompass pose prediction, virtual screening, affinity ranking, and affinity prediction. Virtual screening emerges as a useful paradigm for early drug discovery owing to the availability of high-resolution target structures and ultra-large libraries of compounds [3]. The process of bringing a drug to market is arduous and financially demanding, with a notable failure rate. As a result, drug development prioritizes any approach that can expedite the process and minimize the failure rate. However, the effectiveness of the virtual screening process has been questioned, with researchers suggesting that weaknesses in the modeling of protein-ligand interactions are to blame for less favorable results [4]. The advent of AlphaFold [5] has made it easier to obtain high-precision target structures. Advances in protein-ligand interaction modeling are needed to better utilize these structures for drug discovery.

In the past decades, protein-ligand binding affinity prediction task was mainly performed utilizing molecular force fields and some other calculations [6]. These methods, which once relied heavily on hand-crafted feature engineering, have recently been replaced by various end-to-end machine learning algorithms. These algorithms have evolved from early models like Random Forest [1] and Support Vector Machine [7] to Deep Neural Network [8]. These methods mainly use three types of data, including molecular fingerprints, protein sequences, and crystal structures. An example of a molecular fingerprint application is ECIF [9]. However, designing effective manually extracted features requires significant domain knowledge. The protein sequences are capable of providing sufficient information to serve as a reliable data source in CAPLA [10], although there are proteins that are remotely similar and have low sequence similarity [11]. Then, structure-based methods treat protein-ligand complexes as 3D-grids or molecular graphs, such as Pafnucy [12] and IGN [13]. Recently, there has been a growing interest in applying graph neural networks (GNN) to the affinity prediction task due to their superior performance in processing graph-structured data [14]. Molecules can be naturally represented as graphs, making GNN a suitable tool for automated de novo drug design and drug discovery [15].

**Fig 1:**
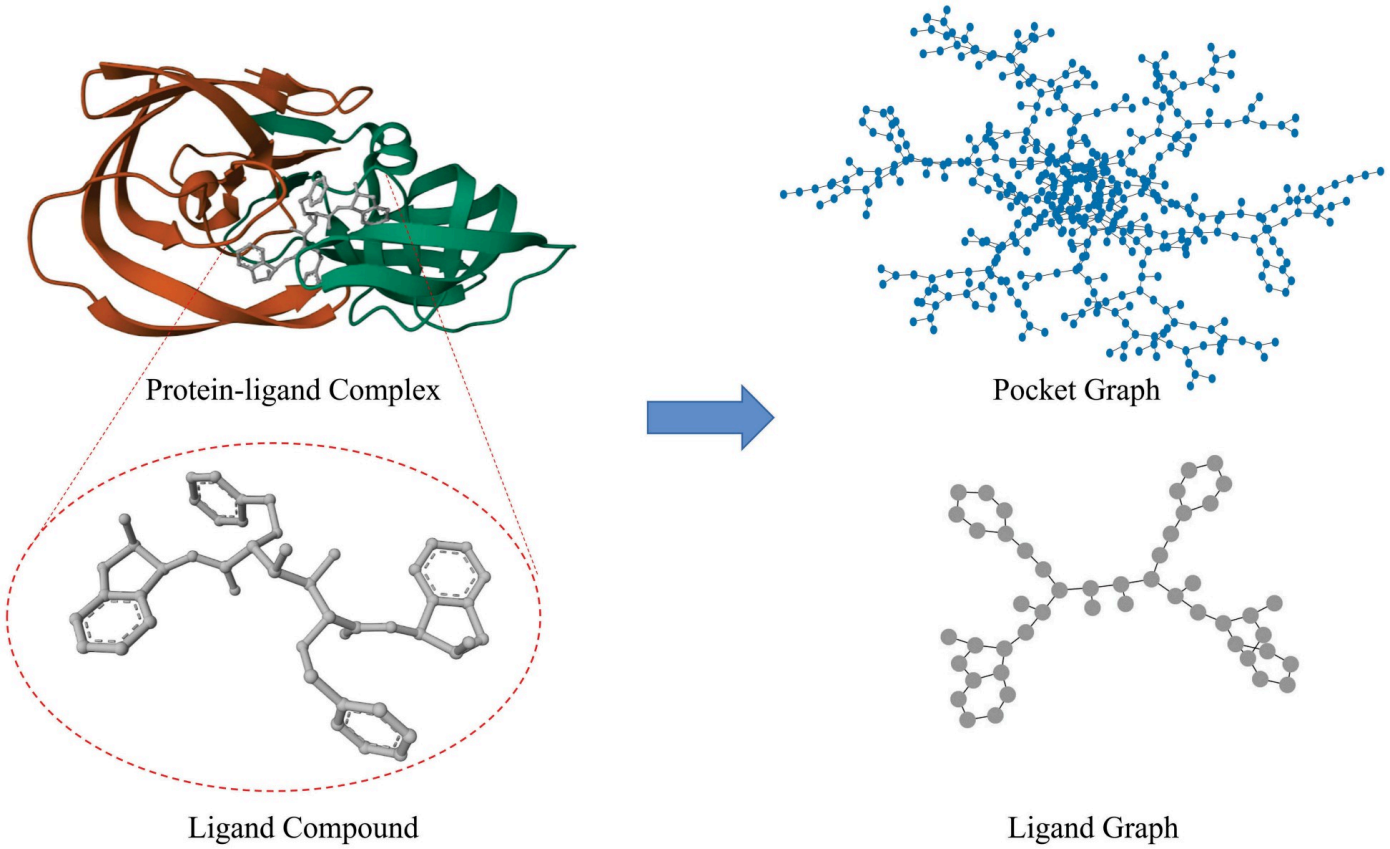
The visualization of 1eby in PDBbindv2016 after conversion. The protein pocket (blue graph) has 513 nodes and 958 edges, while the ligand (grey graph) has 48 nodes and 106 edges. The large difference in volume between the two can lead to overfitting and overlooking the ligand information if they are treated in the same way.

Most existing methods do not take into account the data heterogeneity and imbalance between proteins and ligands, in particular the large discrepancy in volume, as shown in Fig 1. In conventional virtual screening, the protein pocket, even if a mere fraction of the entire protein, commonly includes hundreds of nodes. Conversely, the small ligand typically involves only a few dozen nodes. The presence of such a concern has yet to be observed within other domains of GNN implementation. For protein-protein interaction and drug-drug interaction, all samples are of the same type. However, the existence of a volume gap may have an impact on the protein-ligand affinity prediction. The potent fitting capability of Neural Network may contribute to the overfitting of protein structure. Some researchers have found that adding additional ligand information can improve performance [16], but they only use the RDKit molecular descriptors. We extract the information from the structure of the ligand by graph representation learning to achieve a better description of the ligand. The fact that protein similarity could affect the affinity prediction has also been proved [17]. This supports the assumption that machine learning models place more emphasis on proteins than ligands. Virtual screening can be divided into structure-based virtual screening and ligand-based virtual screening, which means the ligand itself could be a crucial factor in the success of drug discovery. Although fingerprints have established themselves as powerful tools for Gradient Boosting Decision Tree, they have not been widely used in Neural Network.

Based on the above considerations, we developed a new graph neural network-based fusion model with additional ligand feature extraction. To process the transformation of protein-ligand complexes into graphs, the original feature vectors for vertexes and edges were mainly based on the properties of atoms and chemical bonds, such as the type of atom and if the atom is aromatic, which is shown in Table 1. Meanwhile, in order to add more ligand information to counterbalance the data imbalance, feature vectors for vertexes were designed with a relatively more macroscopic perspective for processing the transfer of ligands [18]. To emphasize the notion of heterogeneity, LGN removed protein nodes during the processing of the ligand graph, since the goal was to obtain local features of the protein-ligand complex. The procedure was repeated for batches of complex graphs and ligand graphs, producing the molecular descriptors in the form of vectors that embedded information about the structure. Using this framework, we have generated fingerprints of protein-ligand interactions that can be further processed in our architecture. To this end, we conducted experiments to verify that the fusion model could produce a better result than a single model. We also demonstrated that adding molecular interaction fingerprints could be helpful. Next, we proposed a new method to analyze the model based on the number of training samples and the similarity of the training and test sets. Since the effect of similarity cannot be ignored, a boosting framework was designed to improve the robustness of our model. Through lots of trials, we evaluated the stability and generalization capabilities of the model. Ultimately, the superiority of our model was proved in comparison to various previously published models.

**Table 1:** Brief description of the featurization of complex.

Feature	Description
*The*_*type*_*of*_*atom*	‘C’, ‘N’, ‘O’, ‘S’, ‘F’, ‘P’, ‘Cl’, ‘Br’, ‘I’, ‘B’, ‘Si’, ‘Fe’, ‘Zn’, ‘Cu’, ‘Mn’, ‘Mo’, ‘Other’
*The*_*degree*_*of*_*atom*	0–5
*The*_*formal*_*charge*_*of*_*atom*	the real value
*The*_*chirality*_*of*_*atom*	‘R’, ‘S’, ‘Other’
*The*_*total*_*number*_*of*_*Hs*_*of*_*atom*	0–4

## Materials and methods

### Dataset preparation

In the context of GNN, the accuracy of three-dimensional crystal structures of proteins and ligands is paramount in converting raw data into graph format. Since our task is to predict the binding affinity, reliable experimental affinity data is imperative. The PDBbind dataset [19] is a meticulously curated collection of three-dimensional structures depicting protein-ligand complexes acquired from the Protein Data Bank [20]. Each complex in the dataset is accompanied by an experimentally determined binding affinity. We have primarily leveraged the PDBbind database of version 2016, also known as ‘PDBbindv2016’. PDBbindv2016 is comprised of a ‘general set’ that comprises all the protein-ligand structures in the database, and a ‘refined set’ that is a high-quality subset selected from the general set. In order to validate our models, we utilized the PDBbindv2016 ‘core set’, also known as the ‘CASF-2016 set’, as our test set [21]. The core set is a diverse and non-redundant set of 285 protein-ligand complexes in 57 clusters. Due to its well-balanced and all-encompassing attributes, CASF-2016 serves as a remarkable indicator to assess the predictive capabilities of our model.

The PDBbind platform offers a detailed record of each complex, comprising an experimental value for the inhibition constant Ki, the dissociation constant Kd, or the half-maximal inhibitory concentration IC50. We consider all values to be interchangeable. To comprehensively evaluate the model performance, and following most related works [22, 23], we employed three metrics including the Pearson correlation coefficient (Rp), Root Mean Square Error (RMSE), and Mean Absolute Error (MAE).

$$\begin{array}{ccc} R_{p} & = & \frac{\sum_{i=1}^{n}\left(f\left(x_{i}\right)-\bar{f(x)}\right)\left(Y_{i}-\bar{Y}\right)}{\sqrt{\sum_{i=1}^{n}{\left(f\left(x_{i}\right)-\bar{f(x)}\right)}^{2}}\sqrt{\sum_{i=1}^{n}{\left(Y_{i}-\bar{Y}\right)}^{2}}} \end{array}$$ (1)

$$\begin{array}{ccc} RMSE & = & \sqrt{\frac{1}{N}\sum_{i=1}^{n}{\left(Y_{i}-f\left(x_{i}\right)\right)}^{2}} \end{array}$$ (2)

$$\begin{array}{ccc} MAE & = & \frac{1}{N}\sum_{i=1}^{n}|Y_{i}-f\left(x_{i}\right)| \end{array}$$ (3)

However, since the CASF-2016 set is relatively outdated, in order to ensure validity, the PDBbindv2020 is selected as supplementary. We followed the same time split as defined in the EquiBind paper [24] in which the training and validation sets were the protein-ligand complex structures deposited before 2019 and the test set was the structures deposited after 2019. After removing a few structures that were unable to process using RDKit from the training set, we had 16967 structures for training, 1885 for validation, and 363 for testing. We a need new test set because the similarity between the training set and the test set could be a key factor for the performance. Some of the previous methods might have been validated or trained on a subset of the core set and thus, report optimistic quality numbers. By using a test set consisting solely of recently discovered complexes, while the training and validation sets exclusively used older complexes, we aimed to provide a more representative picture of the average complex encountered in real-world applications. In the realm of new drug discovery, it is customary to employ previous samples to predict the outcomes of new samples, despite the presence of overlap. This practice introduces an element of randomness to the process.

### The fusion model

There are early precedents for the use of fusion models in drug affinity prediction development. For example, combining the learning of 3D convolutional neural networks and spatial graph convolutional neural networks could capture different feature representations of atoms [23]. Small chemical molecules are a rich source of information. Therefore, an additional characterization method was applied to the ligand structure to provide a more comprehensive analysis. To minimize computational cost and maximize efficiency, the protein structure was not treated in the same way. The advantage of graph neural networks is that they are suitable for processing non-Euclidean data structures such as protein pockets and ligand small molecules, and automatically extract structural features, thus obtaining richer information than the usual feature extraction methods [25]. The ultimate goal of graph neural networks is to obtain a vector representation of the whole graph.

Virtual screening can be divided into ligand-based virtual screening (LBVS) [26] and structure-based virtual screening (SBVS) [27]. SBVS relies on the three-dimensional structure of the target and its active site to study the interaction pattern between the receptor and the ligand. With the development of structural biology, molecular biology, and chemical informatics, SBVS has become the mainstream of virtual screening. Its related technologies mainly include molecular docking, receptor-based pharmacophore [28], molecular dynamics simulation [29], etc. In contrast, LBVS generally does not consider the protein structure and instead focuses on analyzing the properties of ligands. Therefore, we believe that the inclusion of additional ligand information in the model may improve the generalization ability of the model.

**Fig 2:**
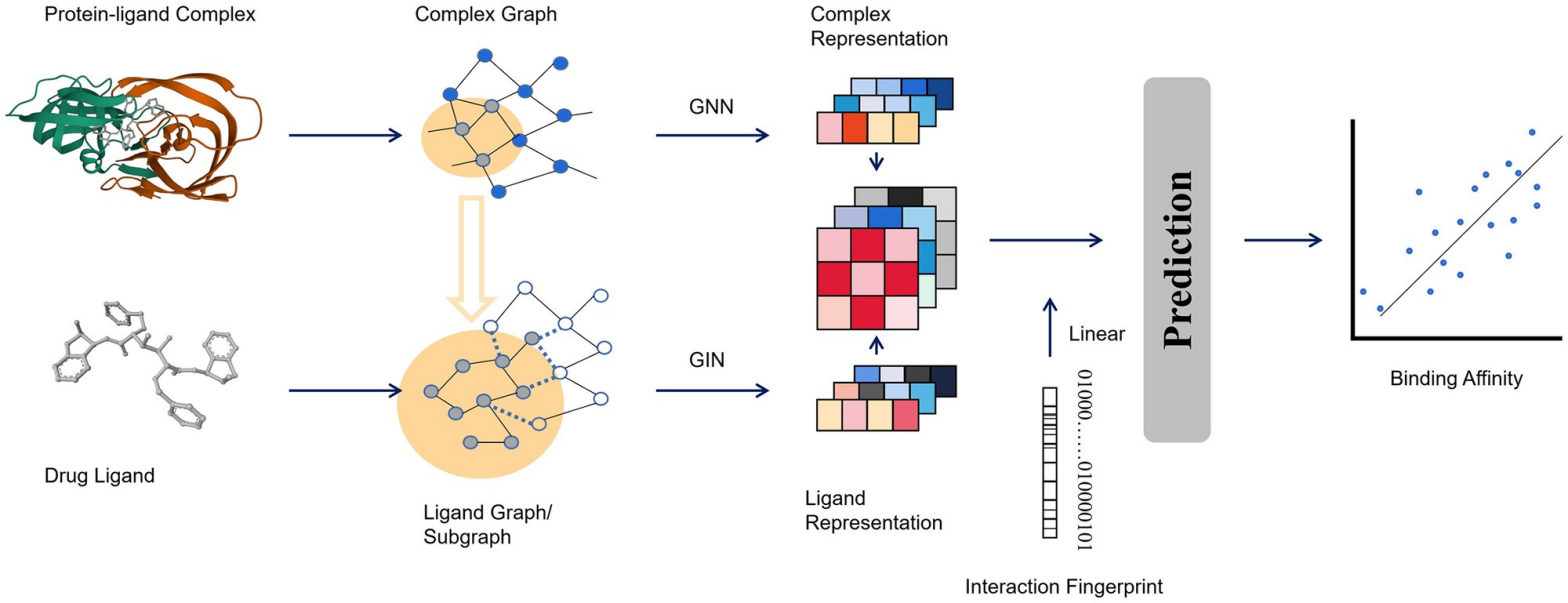
The architecture of the fusion model. The fusion model is mainly composed of three different components. By using the same protein-ligand complex from PDBbind, the different components extract various information and obtain a fixed vector for each. The vectors are concatenated to produce the final prediction. The complementary information improves the performance of the model and makes the outputs more precise. The final outputs are compared with the experimentally measured values.

Based on these observations, we introduced a proficient fusion framework for deep graph learning. To represent a protein-ligand complex, we employed two molecular graphs, namely the complex graph and the ligand graph. According to what has been published [13], in the graphs composed of protein-ligand complexes, the characteristics of nodes and edges were treated in a general way. Node feature vectors represented the atoms’ properties, while edge information depicted the connections between the nodes to signify the graph’s structure. The attention mechanism and the gate recurrent unit were used for complex graphs. The application of the attention mechanism in graph [30] was first introduced in 2018. The gated recurrent unit, originally designed for recurrent neural networks, was also widely used in GNN. In the graphs composed of ligands, the nodes were processed using different characterization methods. The structural information in the graph was extracted by reference to the Graph Isomorphism Network (GIN) architecture [31]. GIN is a powerful graph neural network architecture that in principle implements an upper limit for the solution of the Weisfeiler-Lehman graph isomorphism problem. For each protein-ligand complex within the dataset, the data was utilized twice and subjected to specific characterization techniques to generate two distinct graph structures. These structures were then inputted into separate graph neural networks in order to extract a fixed-length vector for each. The vector read out from the complex graph was called the complex feature, while the vector read out from the ligand graph was called the ligand feature. The architecture of our model is presented in Fig 2.

$$\begin{array}{ccc} Feature\_Complex & = & GNN(Graph\_Complex) \end{array}$$ (4)

$$\begin{array}{ccc} Feature\_Ligand & = & GIN(Graph\_Ligand) \end{array}$$ (5)

The resulting vectors were later combined and a single linear layer mapping was used to attain the final outputs. More details about the hyperparameters and the environment could be seen in Tables 2 and 3.

$$\begin{array}{ccc} Feature & = & Concat(Feature\_Complex,Feature\_Ligand) \end{array}$$ (6)

$$\begin{array}{ccc} Outpout & = & Linear(Feature) \end{array}$$ (7)

**Table 2:** Brief description of the hyperparameters of model.

Hyperparameter	Description
*maximum*_*num*_*epochs*	200
*lr*	0.0001
*w*_*d*	0.000001
*batch*_*size*	128
*dropout*	0.1
*GNN*_*layer*	4
*GIN*_*layer*	3

**Table 3:** Brief description of the environment.

Environment	Description
*GPU*	NVIDIA Geforce RTX 3070Ti
*CPU*	12th Gen Intel(R) Core(TM) i9–12900H
*Python*_*version*	3.7
*PyTorch*_*version*	1.12.1+cu116
*CUDA*_*version*	11.6

In the design of graph neural networks for ligands, the graph attention network and graph transformer network [32] were found not as effective as GIN, as Table 4 showed. This observation could be attributed to the simplicity of the ligand graph structure and the simplicity of feature construction compared to the complex. Consequently, these ligands may not be suitable for processing with attention mechanisms. This highlights the fact that achieving better results does not solely depend on the complexity of the model, but rather on the appropriateness of the fit. This may be due to the characteristics of the graph neural network itself. There are some limitations of graph neural networks. For example, the models tend not to be made very deep and large due to the limitations of graph neural networks themselves, such as the problem of oversmoothing. Some techniques that are more applied in other fields can not be well applied in the graph neural network field. In addition, there is an upper limit to the expressive power of graph neural networks, which is the graph isomorphism problem. According to the conformational relationships in biology, there is a strong correlation between structure and function for ligand small molecules. Therefore, GIN can be used to maximize the extraction of ligand features and applied to the prediction of function. The loss function considered includes MSELoss, SmoothLoss and self-designed loss functions. With Rp and RMSE as metrics, the self-designed loss function is a combination of the two. We found that MSELoss showed the best result.

$$\begin{array}{ccc} MSELoss & = & \frac{1}{N}\sum_{i=1}^{n}{\left(Y_{i}-f\left(x_{i}\right)\right)}^{2} \end{array}$$ (8)

$$\begin{array}{ccc} SmoothL1Loss & = & \frac{1}{N}\sum_{i=1}^{n}\{\begin{array}{cc} 0.5\times {\left(Y_{i}-f\left(x_{i}\right)\right)}^{2}, & \text{if}\;|Y_{i}-f\left(x_{i}\right)|<1\text{,}\\ |Y_{i}-f\left(x_{i}\right)|-0.5, & \text{otherwise} \end{array} \end{array}$$ (9)

$$\begin{array}{ccc} MyLoss & = & \alpha \times R_{p}+\left(1-\alpha \right)\times RMSE \end{array}$$ (10)

**Table 4:** Performance of the different models for ligand.

Model	Dataset	Rp	RMSE	MAE
*GIN*	PDBbindv2016	0.822	1.333	1.063
*GAT*	PDBbindv2016	0.794	1.424	1.128
*GTN*	PDBbindv2016	0.767	1.481	1.180

GIN performs best for the data mining of ligand structure information.

### Fingerprint additions

Apart from the above two methods for extracting overall structure-based features using GNN, additional information on molecular fingerprints was added. Molecular fingerprints are descriptors commonly used to describe the properties of small molecules, and can also be applicable to protein-ligand interaction [33]. Molecular fingerprints incorporated in our model include Simple Ligand–Receptor Interaction Descriptor(SIFP) [34], Extended Connectivity Interaction Features(ECIF) [9] and Circular Fingerprints (CFP) [35]. The first two are applied to protein-ligand interaction and the last is used to describe ligand properties. SIFP can be obtained from the binary interaction fingerprints (IFPs) by summing up the bits corresponding to identical amino acids. This results in a vector of 168 integer numbers corresponding to the product of the number of entries (20 amino acids and one cofactor) and 8 interaction types per amino acid (hydrophobic, aromatic face to face, aromatic edge to face, H-bond donated by the protein, H-bond donated by the ligand, ionic bond with protein cation and protein anion, and interaction with metal ion). The ECIF method encompasses a collection of protein-ligand atom-type pair counts. These counts are determined by considering the connectivity of each atom and thereby establishing the various pair types. ECIF has been successfully utilized in random forests, yielding favorable outcomes. CFP refers to the representation of molecular structures based on atom neighborhoods. This encoding technique allows for the inclusion of a significant amount of local structural information present in a molecule.

Although appearing early, molecular fingerprints continue to be used for small molecule property prediction tasks due to their powerful characterization and interpretation capabilities [36]. Protein-ligand interaction fingerprints are powerful for the analysis and assessment of docking poses to improve docking performance in virtual screening. Each part of molecular fingerprints is based on a biochemical theory and is therefore highly interpretable. Fingerprints are often perceived as being sparsely populated with numerous zero values, rendering them more appropriate for utilization in random forests rather than neural networks. In contrast, the embeddings generated by graph neural networks are condensed, with each value offering valuable information. We regard the latter as a novel form of fingerprints, acquiring insights from the inherent structure of raw data rather than relying on handcrafted features.

### Varying training datasets

In regards to classical methods such as AutoDock Vina [37], it has been observed that the augmentation of the training set does not have a substantial impact on the performance of these models [38]. But machine learning methods are considerably sensitive to the training set and the results of experiments can be significantly influenced by the quality and size of the datasets utilized [39]. For a model with poor generalization performance, its performance may vary dramatically across different distributions of the training set, whereas if a model performs consistently across all distributions of the training set, then it can be shown that the model has good generalization performance. To ensure a comprehensive analysis, the general set was subjected to certain filtering rules to produce three datasets with different sizes. The test set was fixed as CASF-2016, and a ten-fold cross-validation operation was performed on these three training sets. The samples in the smallest dataset are all from the refined set, with 3358 training samples and 374 validation samples. By removing some inaccuracies and IC50 data from the general set, we get the medium size dataset, with 7025 training samples and 781 validation samples. Then the biggest dataset consists of all data in the general set, with 11517 training samples and 1280 validation samples.

In addition to the size of the training set, the distribution of the training set also has a large impact on the results. Recently, a lot of studies have verified the bias hidden in several well established datasets, such as PDBbind, and therefore, it is necessary to determine whether the good performance of model is mainly contributed from the data mining or the hidden bias [40]. Li et al. [17] conducted a study on the impact of similarity on the performance of machine-learning-based scoring functions for protein-ligand interactions. They found that similarity plays a crucial role in the performance of models such as RFscore. Additionally, the authors observed that models trained on dissimilar samples did not outperform classical scoring functions. To investigate this further, the authors divided the test and training sets from PDBbindv2007 based on protein structure and sequence similarity. As the cutoff for similarity increased, the number of training sets decreased and the performance of the models, measured by Rp, also decreased. The authors also noted a significant decrease in ligand similarity when protein structure or sequence similarity decreased, which aligns with previous studies [41]. Furthermore, to assess the model’s ability to generalize, various studies have observed that excluding proteins from the training set that are similar to those in the test set leads to a significant decline in the performance of the scoring function [18]. However, another study has discovered that when nearly identical ligands (with a Tanimoto similarity above 0.9) are excluded from the training data, this correlation actually becomes stronger [16]. It is worth noting that these studies rely on older datasets, which are relatively small. Smaller datasets may not accurately reflect the complexities of the problem, particularly in the context of deep learning. Some methods do not keep the size of the training set constant while removing the parts of the training set that are similar to the test set, and then the decrease in model effectiveness cannot be fully attributed to the removal of similar data.

Based on the available reports, we conducted a new assessment of how the similarities affect the performance of our model. To effectively determine the effect, the similarity of protein sequences, ligand molecular fingerprints and interaction fingerprints were considered separately. The similarity of protein sequences was compared using the Biopython package, and the similarity of ligand molecular fingerprints and the similarity of interaction fingerprints were compared with the Tanimoto coefficient using the Open Drug Discovery Toolkit (ODDT) [42]. In order to treat about one-third of the total 3887 data as similar data, protein sequences with similarity greater than 0.9 were considered as similar data, about 1373. Ligand molecular fingerprints with similarities greater than 0.4 were considered similar data, with approximately 1276. Interacting molecular fingerprints with similarity greater than 0.65 were considered as similar data, and there were about 1225 of them. The number of final training data set used is always 3000, sampled from similar data and dissimilar data, respectively. Similarity is defined as the proportion of similar data to the total, which are 0, 20%, 40%, 60%, 80%, and 100%. That is, for a similarity of 40%, there are 1200 similar data and 1800 dissimilar data in the dataset for training.

## Results

In this section, an ablation study was firstly conducted to ascertain the necessity of adding extra ligand information extracted through GIN. Then, we described and discussed the evaluation results of three different fingerprint additions and their combinations. Following extensive evaluation, the most robust model was identified and trained on several different sized and distributed datasets. In line with PDBbindv2016, the latest version of PDBbind was employed to evaluate the model’s ability. The discussion emphasized the stability and generalization of the model for each performance test.

### Ablation study

Previous study shows that AI model could achieve similar performance even on datasets containing only ligand structures or only protein structures [40] because of its powerful fitting ability. So it’s important to do experiments of training on the free ligands (ligand alone) and the free proteins (protein alone), so we can understand what the AI models learned from the complex structures. Furthermore, the inclusion of an ablation study is imperative for a fusion model. In many instances within machine learning, we construct models comprising diverse components that collectively impact the overall performance. Hence, it becomes crucial to establish methods for assessing the individual contributions of these components towards the holistic model. This is precisely where the notion of an ablation study emerges, wherein specific segments of the network are deliberately eliminated to enhance our comprehension of the network’s behavior.

In order to assess the fusion approach, two investigations were carried out. Firstly, we aim to determine if the fusion model effectively amalgamates the unique insights gleaned from the two GNN models to generate improved predictions in comparison to the individual model. Secondly, we endeavor to determine the rational method of combining the information to achieve optimal performance.

In Table 5, the Rp between the predicted and experimental affinities attained by each model, as well as the RMSE and MAE, are presented. The results indicate that utilizing both complex and ligand simultaneously leads to better outcomes. However, implementing solely complex exhibits a higher Rp value and faster convergence speed than using only ligand. This can be attributed to the fact that complexes possess more intricate structures and richer feature information. In the complex graph, each node is equipped with a 41-length feature, and each edge has a 21-length feature. Conversely, in the ligand graph, every node only has a 27-length feature with no edge feature, which justifies the inferior performance of the ligand-only approach. Nevertheless, exclusively utilizing the ligand results in better stability, leading to enhanced generalization ability. Utilizing the principles of SAR (Structure-activity relationship), employing solely the structural and chemical ligand data can yield a more favorable outcome rather than a haphazard consequence. In the case where the ligand feature has a length of 16 and the complex feature has a length of 128, the individual model demonstrates the most optimal outcomes separately. However, when considering the fusion model, the situation becomes more intricate, prompting us to employ various methods in order to regulate the variables effectively. Through a comparative analysis of fusion-1 to fusion-6 models, it becomes evident that the model’s performance consistently exceeds 0.810 when the ratio of complex feature to ligand feature is equal to 1. Interestingly, despite fusion-7 and fusion-1 possessing the same length of ligand feature, the inclusion of more extensive complex features does not result in improved outcomes. Through empirical testing, it has been found that a specific combination can substantially enhance the overall result. The result shows that the incorporation of complementary ligand information is essential for predicting the binding affinity of the proposed fusion model.

**Table 5:** Performance of the individual model and the fusion model.

Model	Dataset	Full Length	Description	Rp	RMSE	MAE
*Complex* − 1	PDBbindv2016	1	Only Complex	0.797	1.442	1.153
*Complex* − 2	PDBbindv2016	16	Only Complex	0.786	1.424	1.127
*Complex* − 3	PDBbindv2016	32	Only Complex	0.776	1.404	1.120
*Complex* − 4	PDBbindv2016	64	Only Complex	0.794	1.412	1.148
*Complex* − 5	PDBbindv2016	128	Only Complex	0.807	1.407	1.148
*Complex* − 6	PDBbindv2016	256	Only Complex	0.795	1.417	1.113
*Ligand* − 1	PDBbindv2016	1	Only Ligand	0.714	1.604	1.286
*Ligand* − 2	PDBbindv2016	16	Only Ligand	0.765	1.47	1.174
*Ligand* − 3	PDBbindv2016	32	Only Ligand	0.760	1.493	1.196
*Ligand* − 4	PDBbindv2016	64	Only Ligand	0.745	1.497	1.205
*Ligand* − 5	PDBbindv2016	128	Only Ligand	0.760	1.448	1.160
*Ligand* − 6	PDBbindv2016	256	Only Ligand	0.742	1.474	1.180
*Fusion* − 1	PDBbindv2016	32	Complex: Ligand = 16:16	0.812	1.388	1.113
*Fusion* − 2	PDBbindv2016	64	Complex: Ligand = 32:32	0.817	1.318	1.035
*Fusion* − 3	PDBbindv2016	128	Complex: Ligand = 64:64	0.812	1.296	1.028
*Fusion* − 5	PDBbindv2016	256	Complex: Ligand = 128:128	0.811	1.371	1.109
*Fusion* − 6	PDBbindv2016	512	Complex: Ligand = 256:256	0.819	1.257	0.989
*Fusion* − 7	PDBbindv2016	48	Complex: Ligand = 32:16	0.806	1.387	1.115
*Fusion* − 8	PDBbindv2016	80	Complex: Ligand = 64:16	0.810	1.384	1.110
*Fusion* − 9	PDBbindv2016	144	Complex: Ligand = 128:16	0.816	1.490	1.199
*Fusion* − 10	PDBbindv2016	272	Complex: Ligand = 256:16	0.805	1.403	1.125
*Fusion* − 11	PDBbindv2016	160	Complex: Ligand = 128:32	0.822	1.333	1.063
*Fusion* − 12	PDBbindv2016	192	Complex: Ligand = 128:64	0.805	1.601	1.314
*Fusion* − 13	PDBbindv2016	384	Complex: Ligand = 128:256	0.819	1.287	1.026

**Fig 3:**
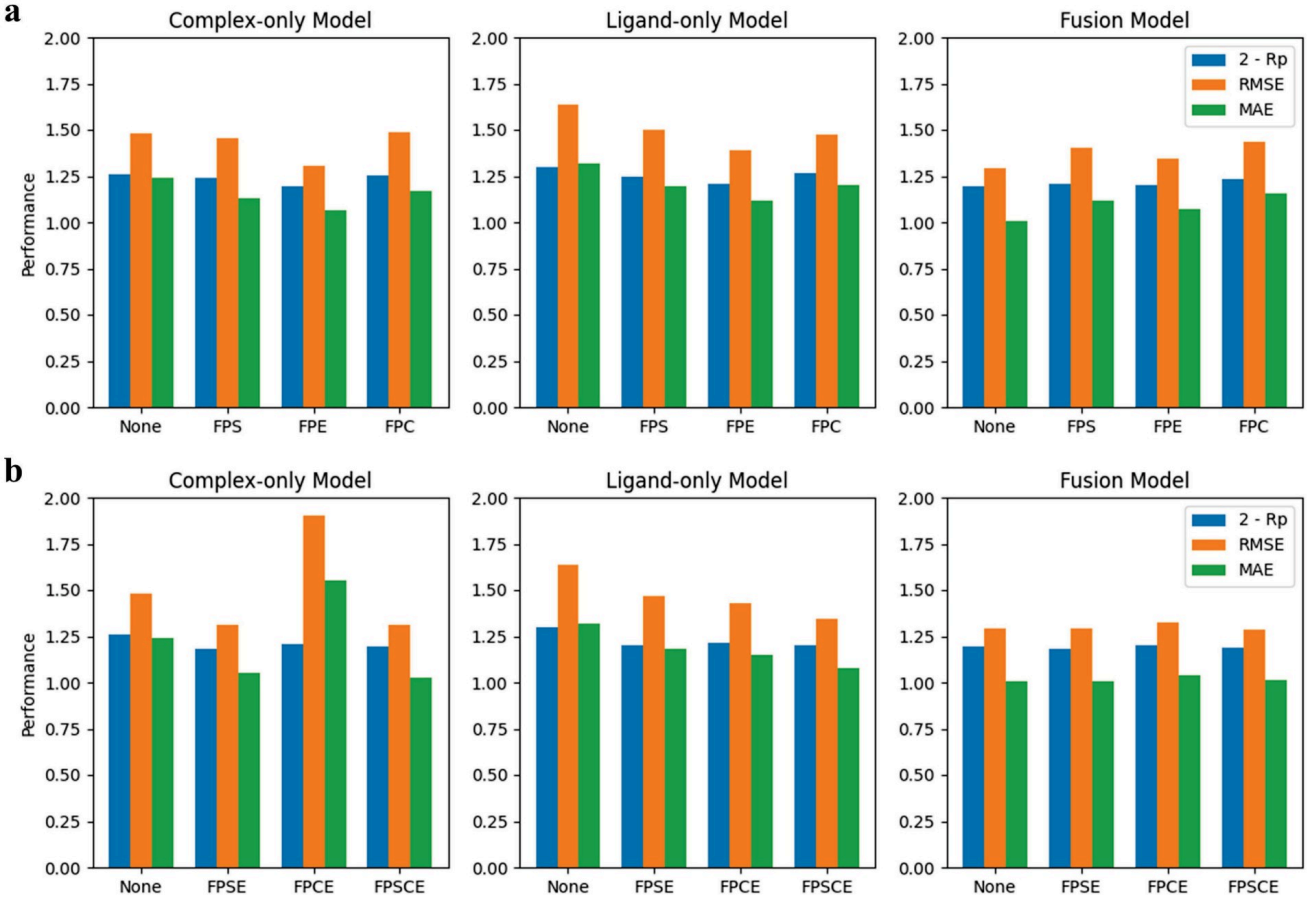
The visualization of the effects of different fingerprints and the performance of different models. Notably, higher values of Rp correspond to superior results, and we use 2-Rp in the diagram to facilitate comparison. For optimal results, all three metrics should exhibit low values. FPS denotes fingerprint SIFP, FPE denotes fingerprint ECIF, FPC denotes fingerprint CFP and FPSE denotes the combination of SIFP and ECIF.

To enhance the feature richness of our input, molecular fingerprints were incorporated in this study. In order to exclude the impact of low-quality data, we used the refined set of PDBbindv2016 as the training set. As depicted in Fig 3, our results showcase that, in most cases, a model that combines structure-based feature with interaction fingerprints performs better than a corresponding model that only utilizes structure-based feature. The first fingerprint considered was SIFP with a length of 168, which showed improvement for both the complex-only and ligand-only models. It can be seen that the addition of even the simplest fingerprints of interacting molecules can enhance the effect. However, the combination of the Fusion model and SIFP didn’t enhance the effect. Our study then examined the ECIF fingerprint of length 1746, which greatly enhanced the results even more than SIFP for these two models, but still made the fusion model perform worse. It is important to highlight that not all molecular fingerprints contribute to improved outcomes. Specifically, the molecular property descriptor CFP, which has a length of 2048, did not significantly enhance the results in most scenarios, except when only the ligand is utilized. However, it is crucial to note that this approach may exacerbate the tendency of overfitting. For our model, it’s obvious that molecular interaction fingerprints are always superior to ligand molecular fingerprints alone.

The majority of combinations demonstrated superior efficacy over a singular molecular fingerprint. Notably, the molecular fingerprints were merged with the GNN results at the end after transformation through only one linear layer. Our study showed that the use of multiple linear layers for the molecular fingerprints led to an increase in the overfitting of the model. Almost all combinations were better than one molecular fingerprint alone, and the best one was the combination of SIFP and ECIF, which was called FPSE. Surprisingly, the combination of just the complex and molecular fingerprint SE was better than adding ligand information. However, this aspect of the study only considered correlation coefficients, and the comparison was conducted on a relatively small dataset. Therefore, it would be ill-advised to draw conclusions on which model worked best based solely on the above results. By comparing the fusion model and the combination of complex model and fingerprint CFP, it becomes evident that the embedding derived from the ligand’s structure through GIN contains a more abundant array of information and possesses superior representation capabilities when compared to the ligand molecular descriptor.

### Model and its generalization

**Fig 4:**
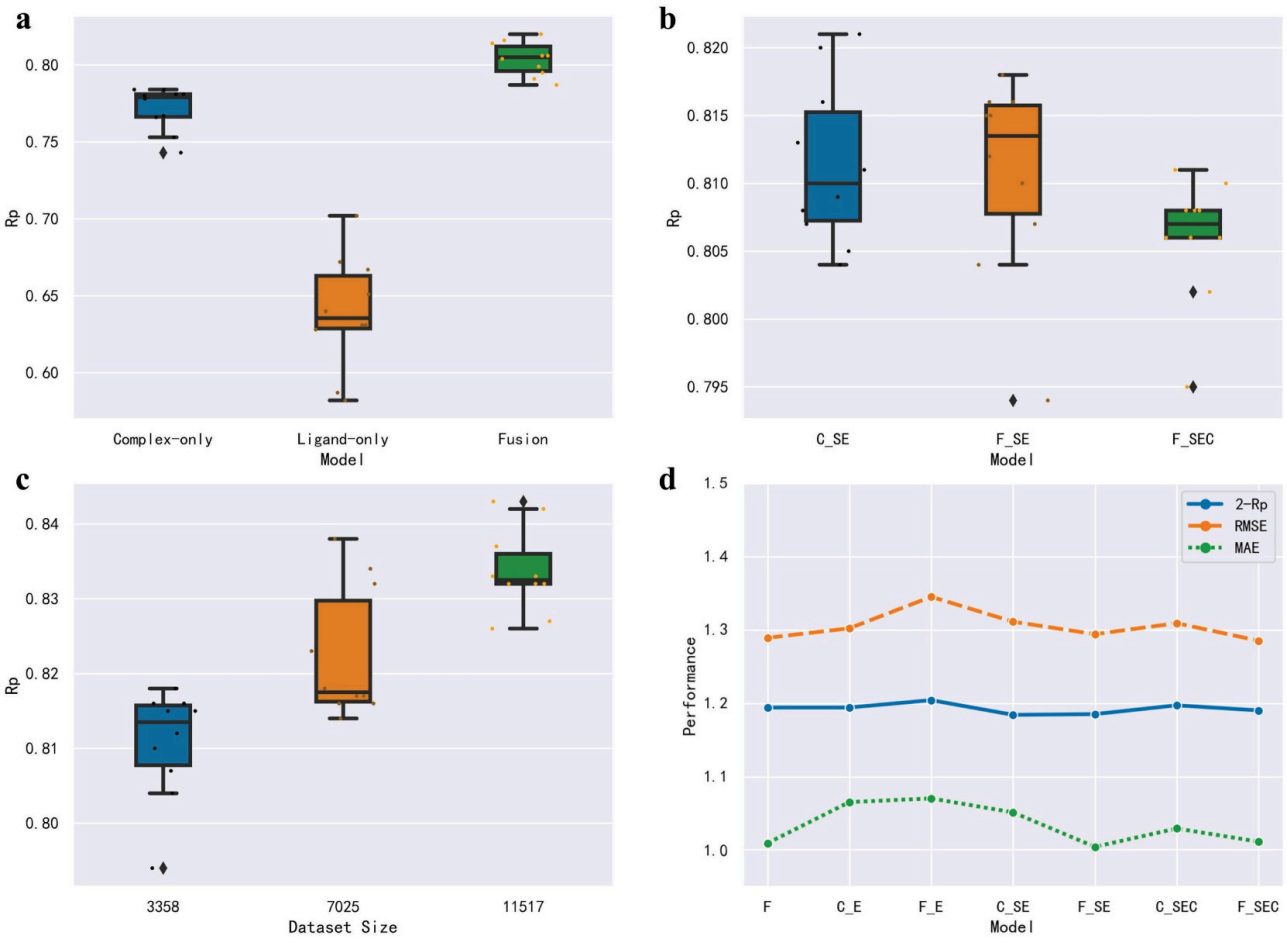
The analysis of different models. Subplots a, b and c represent the ten-fold cross-validation of the different models and different dataset sizes. F denotes the Fusion model, C E denotes the Complex-only model with fingerprint ECIF. Subplot d shows the three metrics for all preferred models. In subplot c, the medium dataset doesn’t use samples that have records of IC50 while the biggest dataset uses all the data, which means Ki, Kd and IC50 are interchangeable.

Fig 4 presents a range of models that have been mentioned or demonstrated relatively favorable results. The model that exhibited a combination of both stability and performance, emerged as the most promising model. In order to better evaluate the different models and understand this result, after obtaining several relatively well-performing models, the data were first divided randomly using ten folds and the model with the best results was selected based on the results of the ten folds. The results show that the best stability should be the model of F_SCE(the combination of fusion model and FPSCE), but the best performance is the model of C_SE(the combination of complex_only model and FPSE). And the one that combines stability and performance is the F_SE model. Considering the complexity of the model and the performance of the molecular fingerprint CFP mentioned before, F_SE was chosen as the final model. C_SE has a higher correlation coefficient but the RMSE is also larger. Taking it into account, we chose F_SE as the final model. The best performance is obtained by combining of our fusion model with FPSE (the combination of fingerprint SIFP and ECIF). This model is also the model used in the final ensemble.

Once the best model is obtained, it is straightforward to evaluate the effect of the model on the 2016 version of the dataset. Generally, while training on the larger general data set could improve performance, it has the drawback of noisier binding affinity measurements and lower-resolution 3D structures. To understand the effect of the datasets on the results, three datasets of different sizes were used with ten-fold validation. A consistent improvement in performance was observed when larger sets were utilized. Based on the Fig 4, it is apparent that augmenting the training data size from 3358 to 7025 leads to remarkable improvements in the model’s performance, as evidenced by a significant increase in the upper limit. When the size is increased to 11517, the model not only shows improvements in performance, but also in stability. Our findings indicate that even the inclusion of low-quality data can enhance model performance.

**Fig 5:**
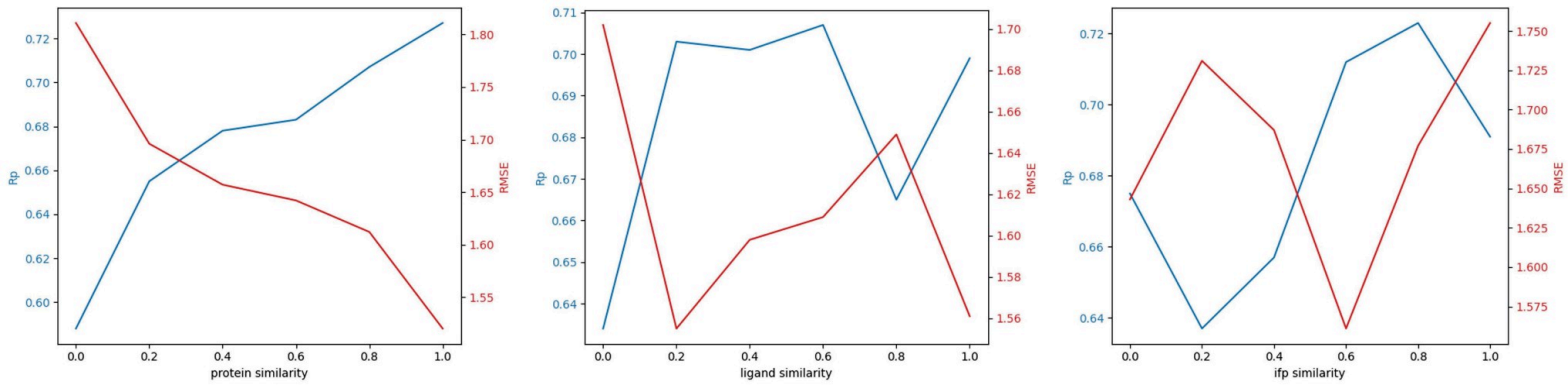
The analysis of similarity. In this plot, similarity is 0 means that all training samples are selected from the subset in which the similarity between the samples and the test set is below a certain value. According to the Pearson correlation coefficient (Rp) and Root mean square error (RMSE) of predicted versus experimental binding affinity, the performance of the model continues to improve as protein similarity increases. The ifp denotes interaction fingerprint.

When the distribution of the model is altered, the performance of the model also varies significantly. In order to assess the stability of our model, the training set is partitioned according to its similarity to the test set, including protein sequence similarity, ligand molecular fingerprint similarity, and interaction molecular fingerprint similarity. When the similarity of two protein sequences is greater than 30%, their tertiary structures as well as biological functions can also be presumed to be very close [43]. Apparently, the predictive power of the model is observed to increase when the protein sequence similarity is higher, as shown in Fig 5. This pattern aligns with the observations made in several previous studies. The similarity of ligands and interaction fingerprints, however, has shown statistically insignificant results. Specifically, the results demonstrate that the model attains optimal performance for ligand fingerprints at a similarity level of 0.6 and for interaction fingerprints at a similarity level of 0.8. Therefore, it is crucial to acknowledge that if the proteins in the complex that are to be predicted differ substantially from those in the training set, the prediction results are likely to be inaccurate. The continuous accumulation of data is necessary to overcome this problem.

**Fig 6:**
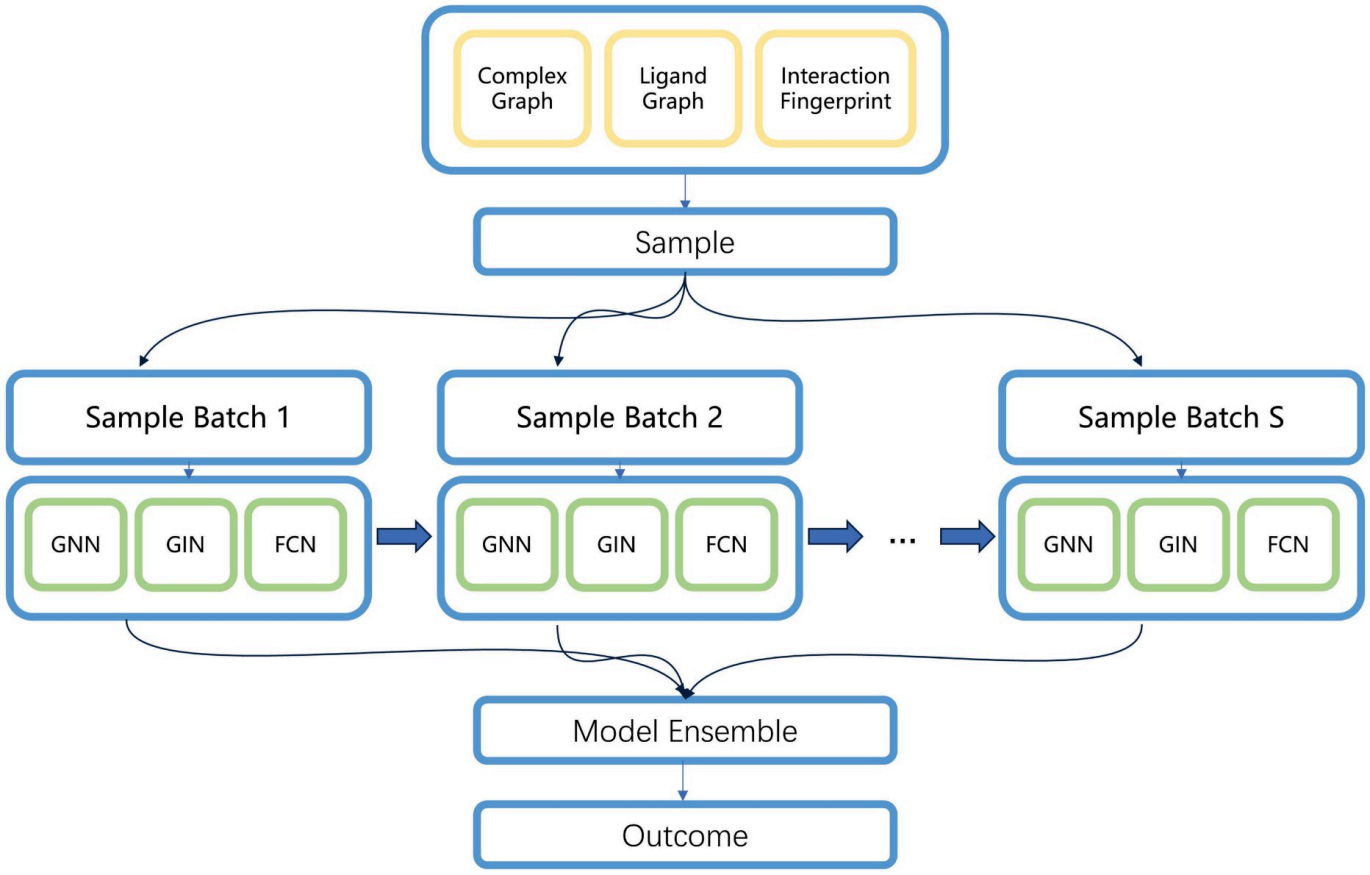
The framework of the ensemble model. By using a boosting framework, we could get a more robust model and better performance. For each model, we only use part of the dataset by random sampling.

Considering the impacts of the dataset similarity that cannot be ignored, we use ensemble learning to get a more robust model and make more stable prediction, which is shown in Fig 6. The combination of GNN and ensemble learning has been used in social bot detection [44]. Multiple models were trained using different subsets of the PDBbindv2016 dataset. The consequences of these models were then ensembled by calculating their mean value. Through this approach, the best result for Rp was obtained, with a value of 0.858 and an ensemble of 40 models.

### Comparsion

**Fig 7:**
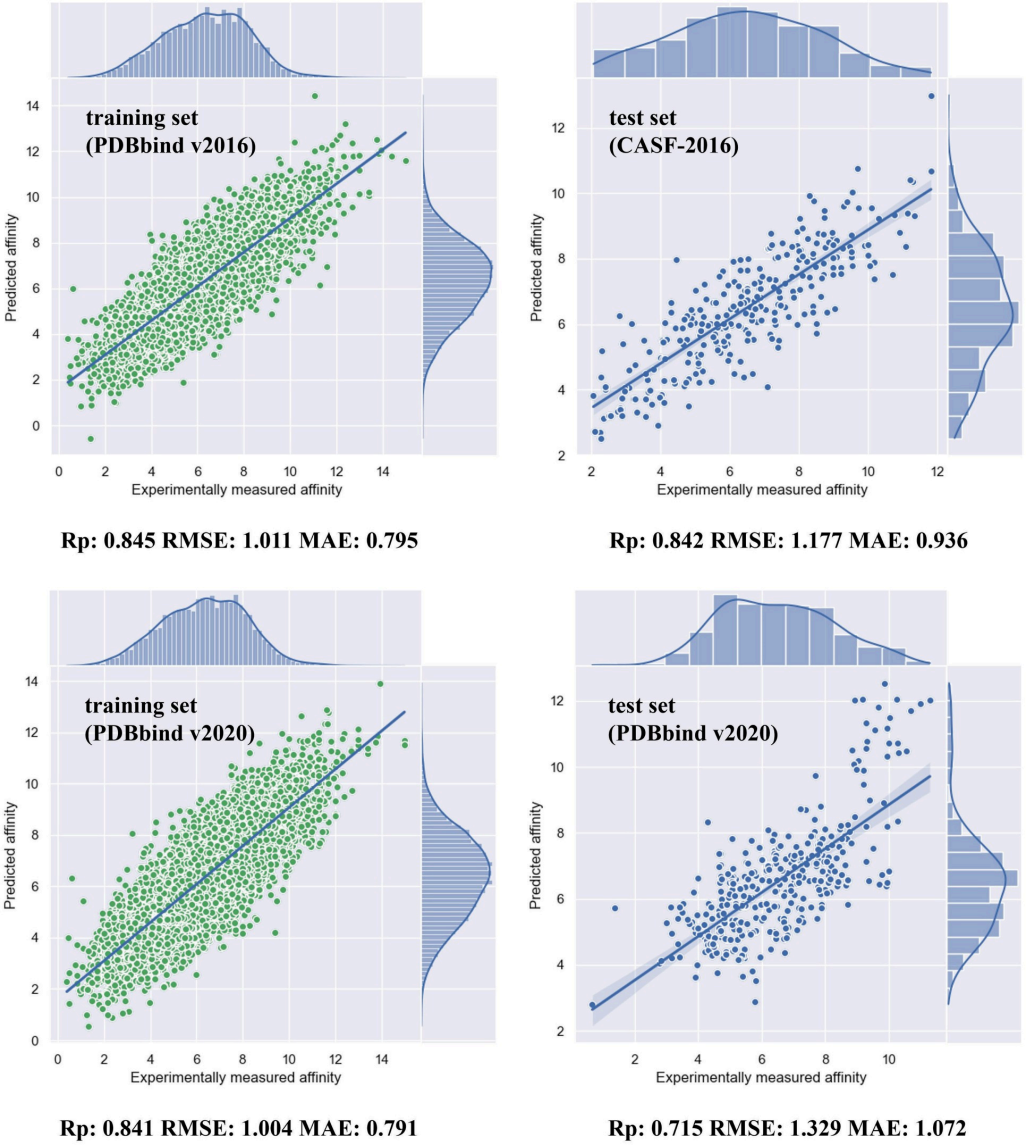
The performance of our fusion model on the training set and test set of two datasets. For both training set, the prediction affinity and experimentally measured affinity are fitted well. For test set, CASF-2016 shows preferable outcome while PDBbindv2020 has more outliers. The reason is that PDBbindv2020 has more novel samples whose structures have just been found. Since the purpose of prediction is to find more hopeful candidate than others, the coefficient is commonly used as metric in this field.

In order to validate the superiority of our single model, it is indispensable to make comparisons with other existing models. For the sake of fairness, we only considered those models that utilized the PDBbindv2016 as training set. We compared our method with four different methods, as Table 6 shows. One of them, FAST [23] is based on 3DCNN and GNN fusion method. OnionNet [22] is a 2DCNN method using features that are distance-based atomic pair counting. RF-Score-v3 [45] is a random forest approach. AGL-score [46] is proposed using multi-scale, multi-class weight coloring for subgraph data representation. GIGN [47] is a geometric interaction graph neural network model that incorporates 3D structures and physical interactions. Our method not only outperforms classical machine learning methods, but is also shown to be superior to the newer deep learning techniques. The scatter plot depicted in Fig 7 shows the outcomes, wherein a fit is observed between the predicted and actual values of both the training set and the test set.

**Table 6:** Comparison on CASF-2016.

Model	Dataset	Rp	RMSE	MAE
*Our* − *method*	PDBbindv2016	0.842	1.177	0.936
*FAST*	PDBbindv2016	0.810	1.308	1.019
*OnionNet*	PDBbindv2016	0.816	1.278	0.984
*AGL* − *Score*	PDBbindv2016	0.833	1.271	NA
*RF* − *Score* − *v*3	PDBbindv2016	0.800	1.390	NA
*GIGN*	PDBbindv2016	0.840	1.190	NA

For a fair comparison, we chose models with the same version of the training dataset.

We then employed the most recent data from PDBbindv2020 as the training set and test set to evaluate the generalization of our approach. After removing a few structures that were unable to be processed from the training set, we had 16967 structures for training. A number of representative methods were selected for comparison, as Table 7 shows. MONN [48] is a sequence-based multi-objective neural network approach. IGN [13] and HOLOPROT [49] are both graph neural network-based approaches. TANKBind [50] is a polar coordinate system-based neural network. By comparing with these methods, it is evident that our model exhibits the lowest root mean square error and a relatively superior Pearson correlation coefficient. The size of the training set used in our method is smaller than that of TANKBind. In addition, our hyperparameters are fixed since using the minimal dataset and these might not be the optimal hyperparameters for the largest dataset. This may explain the reason why our method is slightly inferior to TANKBind in terms of coefficient. TANKBind is based on geometric deep learning and our model shows comparable performance.

**Table 7:** Comparison on PDBbindv2020.

Model	Dataset	Rp	RMSE	MAE
*Our* − *method*	PDBbindv2020	0.715	1.329	1.072
*MONN*	PDBbindv2020	0.624	1.438	1.143
*IGN*	PDBbindv2020	0.698	1.433	1.169
*HOLOPROT*	PDBbindv2020	0.602	1.546	1.208
*TANKBind*	PDBbindv2020	0.726	1.346	1.070

The latest version of PDBbind is used to prove the generalization and the ability to predict recently discovered complexes.

## Discussion

In this paper, we established a new graph neural network-based fusion model with additional ligand feature extraction and interaction fingerprints to predict protein-ligand binding affinity. Although proteins and ligands are two different molecules in nature, most methods only use the same method to characterize them. When building a graph, distinguishing between carbon from proteins and carbon from ligands doesn’t fully capture the difference between the nodes. In contrast, LGN can not only effectively describe the global features of protein-ligand complexes, but also emphasize the subgraph part of the graph with special significance, which is also called the ligand graph. In conclusion, LGN simultaneously learns information from complex graphs and subgraphs and combines the local features with the global features to achieve accurate prediction.

We further confirmed the stability of our model via ten-fold cross-validation and introduced a new method to analyze the impact of the size of the training set and the similarity between the training and test sets. It turns out that the prediction becomes more accurate as more data is used for training, even if the overall quality of the samples becomes low. Furthermore, the prediction is influenced more by protein similarity than by ligand similarity and interaction fingerprint similarity, which supports our initial hypothesis that size disparities between proteins and ligands may lead to overfitting. By using a boosting framework, we improve the robustness and the performance of our model further. The lack of precise protein structure and accurate affinity data are a major limitation to prediction accuracy. To overcome this limitation, future directions could combine structure-based protein-ligand affinity prediction and semantic-based drug-target prediction (DTI) for more efficient drug discovery.

GNN could be applied in all aspects of drug discovery, such as prediction of pocket locations [51], protein-protein interactions [52], and drug-drug interactions [53]. In the other field of computational biology, there is also a large number of important research on interaction prediction. For example, the interaction between long non-coding RNA (lncRNA) and microRNA (miRNA) plays an important regulatory role in many biological processes [54]. Some of these related studies make great progress in effectively utilizing the GNN approach [55, 56] and molecular fingerprint [57]. The advancement of these studies [58–60] would provide valuable insights into protein-ligand affinity prediction.

## Supporting information

S1 Table The effects of different fingerprints and the performance of different models. (DOCX)

S2 Table The analysis of different models. (DOCX)

S3 Table The analysis of similarity. (DOCX)

S4 Table PDB ID of protein-ligand complexes in each test set. (DOCX)
