# Supplementary material for: Improving structure-based protein-ligand affinity prediction by graph representation learning and ensemble learning
Source: PLoS One. 2024 Jan 17;19(1):e0296676. doi: 10.1371/journal.pone.0296676 (PMC10793902; doi:10.1371/journal.pone.0296676)
Supplement: S1 Table — (DOCX) [file pone.0296676.s001.docx]

#### S1 The effects of different fingerprints and the performance of different models.

This is the raw data for Fig. 3.

a

|  | None | FPS | FPE | FPC |
| --- | --- | --- | --- | --- |
| C_Rp | 0.743 | 0.759 | 0.806 | 0.745 |
| C_RMSE | 1.479 | 1.455 | 1.302 | 1.484 |
| C_MAE | 1.240 | 1.127 | 1.065 | 1.170 |

|  | None | FPS | FPE | FPC |
| --- | --- | --- | --- | --- |
| L_Rp | 0.702 | 0.751 | 0.792 | 0.736 |
| L_RMSE | 1.635 | 1.5 | 1.391 | 1.475 |
| L_MAE | 1.316 | 1.195 | 1.116 | 1.199 |

|  | None | FPS | FPE | FPC |
| --- | --- | --- | --- | --- |
| F_Rp | 0.806 | 0.791 | 0.796 | 0.767 |
| F_RMSE | 1.289 | 1.402 | 1.345 | 1.433 |
| F_MAE | 1.009 | 1.12 | 1.070 | 1.155 |

b

|  | None | FPSE | FPCE | FPSCE |
| --- | --- | --- | --- | --- |
| C_Rp | 0.743 | 0.816 | 0.794 | 0.803 |
| C_RMSE | 1.479 | 1.311 | 1.902 | 1.309 |
| C_MAE | 1.240 | 1.051 | 1.555 | 1.029 |

|  | None | FPSE | FPCE | FPSCE |
| --- | --- | --- | --- | --- |
| L_Rp | 0.702 | 0.800 | 0.784 | 0.798 |
| L_RMSE | 1.635 | 1.466 | 1.426 | 1.344 |
| L_MAE | 1.316 | 1.185 | 1.148 | 1.075 |

|  | None | FPSE | FPCE | FPSCE |
| --- | --- | --- | --- | --- |
| F_Rp | 0.806 | 0.815 | 0.799 | 0.810 |
| F_RMSE | 1.289 | 1.294 | 1.325 | 1.285 |
| F_MAE | 1.009 | 1.004 | 1.038 | 1.011 |
