# Supplementary material for: Improving structure-based protein-ligand affinity prediction by graph representation learning and ensemble learning
Source: PLoS One. 2024 Jan 17;19(1):e0296676. doi: 10.1371/journal.pone.0296676 (PMC10793902; doi:10.1371/journal.pone.0296676)
Supplement: S2 Table — (DOCX) [file pone.0296676.s002.docx]

#### S2 The analysis of different models.

This is the raw data for Fig. 4.

a

| index | 0 | 1 | 2 | 3 | 4 | 5 | 6 | 7 | 8 | 9 |
| --- | --- | --- | --- | --- | --- | --- | --- | --- | --- | --- |
| Complex | 0.743 | 0.781 | 0.766 | 0.78 | 0.784 | 0.767 | 0.784 | 0.781 | 0.778 | 0.753 |
| Ligand | 0.702 | 0.628 | 0.64 | 0.667 | 0.651 | 0.582 | 0.672 | 0.631 | 0.587 | 0.631 |
| Fusion | 0.806 | 0.787 | 0.82 | 0.806 | 0.804 | 0.791 | 0.816 | 0.814 | 0.795 | 0.799 |

b

| index | 0 | 1 | 2 | 3 | 4 | 5 | 6 | 7 | 8 | 9 |
| --- | --- | --- | --- | --- | --- | --- | --- | --- | --- | --- |
| C_SE | 0.816 | 0.804 | 0.808 | 0.82 | 0.811 | 0.821 | 0.807 | 0.813 | 0.805 | 0.809 |
| F_SE | 0.815 | 0.794 | 0.815 | 0.807 | 0.816 | 0.816 | 0.804 | 0.81 | 0.812 | 0.818 |
| F_SEC | 0.81 | 0.808 | 0.811 | 0.802 | 0.808 | 0.806 | 0.808 | 0.795 | 0.806 | 0.806 |

c

| index | 0 | 1 | 2 | 3 | 4 | 5 | 6 | 7 | 8 | 9 |
| --- | --- | --- | --- | --- | --- | --- | --- | --- | --- | --- |
| 3358 | 0.815 | 0.794 | 0.815 | 0.807 | 0.816 | 0.816 | 0.804 | 0.81 | 0.812 | 0.818 |
| 7025 | 0.816 | 0.838 | 0.818 | 0.832 | 0.817 | 0.834 | 0.823 | 0.816 | 0.814 | 0.817 |
| 11517 | 0.833 | 0.827 | 0.837 | 0.832 | 0.832 | 0.832 | 0.833 | 0.826 | 0.843 | 0.842 |

d

| index | Rp | RMSE | MAE |
| --- | --- | --- | --- |
| F | 0.806 | 1.289 | 1.009 |
| C_E | 0.806 | 1.302 | 1.065 |
| F_E | 0.796 | 1.345 | 1.07 |
| C_SE | 0.816 | 1.311 | 1.051 |
| F_SE | 0.815 | 1.294 | 1.004 |
| C_SEC | 0.803 | 1.309 | 1.029 |
| F_SEC | 0.81 | 1.285 | 1.011 |
