# Supplementary material for: Improving structure-based protein-ligand affinity prediction by graph representation learning and ensemble learning
Source: PLoS One. 2024 Jan 17;19(1):e0296676. doi: 10.1371/journal.pone.0296676 (PMC10793902; doi:10.1371/journal.pone.0296676)
Supplement: S3 Table — (DOCX) [file pone.0296676.s003.docx]

#### S3 The analysis of similarity.

This is the raw data for Fig. 5.

Protein similarity

| index | 0 | 0.2 | 0.4 | 0.6 | 0.8 | 1 |
| --- | --- | --- | --- | --- | --- | --- |
| RP | 0.588 | 0.655 | 0.678 | 0.683 | 0.707 | 0.727 |
| RMSE | 1.811 | 1.696 | 1.657 | 1.642 | 1.612 | 1.52 |
| MAE | 1.471 | 1.37 | 1.573 | 1.353 | 1.293 | 1.21 |

Ligand similarity

| index | 0 | 0.2 | 0.4 | 0.6 | 0.8 | 1 |
| --- | --- | --- | --- | --- | --- | --- |
| RP | 0.634 | 0.703 | 0.701 | 0.707 | 0.665 | 0.699 |
| RMSE | 1.702 | 1.555 | 1.598 | 1.609 | 1.649 | 1.561 |
| MAE | 1.346 | 1.246 | 1.258 | 1.327 | 1.296 | 1.294 |

Ifp similarity

| index | 0 | 0.2 | 0.4 | 0.6 | 0.8 | 1 |
| --- | --- | --- | --- | --- | --- | --- |
| RP | 0.675 | 0.637 | 0.657 | 0.712 | 0.723 | 0.691 |
| RMSE | 1.643 | 1.731 | 1.687 | 1.561 | 1.677 | 1.755 |
| MAE | 1.335 | 1.399 | 1.341 | 1.274 | 1.347 | 1.429 |
