# Supplementary material for: Improving structure-based protein-ligand affinity prediction by graph representation learning and ensemble learning
Source: PLoS One. 2024 Jan 17;19(1):e0296676. doi: 10.1371/journal.pone.0296676 (PMC10793902; doi:10.1371/journal.pone.0296676)
Supplement: S4 Table — (DOCX) [file pone.0296676.s004.docx]

#### S4 PDB ID of protein-ligand complexes in each test set

| **test set** | **pdb code** |
| --- | --- |
| CASF-2016 | '1a30', '1bcu', '1bzc', '1c5z', '1.00E+66', '1eby', '1g2k', '1gpk', '1gpn', '1h22', '1h23', '1k1i', '1lpg', '1mq6', '1nc1', '1nc3', '1nvq', '1o0h', '1o3f', '1o5b', '1owh', '1oyt', '1p1n', '1p1q', '1ps3', '1q8t', '1q8u', '1qf1', '1qkt', '1r5y', '1s38', '1sqa', '1syi', '1uto', '1vso', '1w4o', '1y6r', '1yc1', '1ydr', '1ydt', '1z6e', '1z95', '1z9g', '2al5', '2br1', '2brb', '2c3i', '2cbv', '2cet', '2fvd', '2fxs', '2hb1', '2iwx', '2j78', '2j7h', '2p15', '2p4y', '2pog', '2qbp', '2qbq', '2qbr', '2qe4', '2qnq', '2r9w', '2v00', '2v7a', '2vkm', '2vvn', '2vw5', '2w4x', '2w66', '2wbg', '2wca', '2weg', '2wer', '2wn9', '2wnc', '2wtv', '2wvt', '2x00', '2xb8', '2xbv', '2xdl', '2xii', '2xj7', '2xnb', '2xys', '2y5h', '2yfe', '2yge', '2yki', '2ymd', '2zb1', '2zcq', '2zcr', '2zda', '2zy1', '3acw', '3ag9', '3ao4', '3arp', '3arq', '3aru', '3arv', '3ary', '3b1m', '3b27', '3b5r', '3b65', '3b68', '3bgz', '3bv9', '3cj4', '3coy', '3coz', '3d4z', '3d6q', '3dd0', '3dx1', '3dx2', '3dxg', '3e5a', '3.00E+92', '3.00E+93', '3ebp', '3ehy', '3ejr', '3f3a', '3f3c', '3f3d', '3f3e', '3fcq', '3fur', '3fv1', '3fv2', '3g0w', '3g2n', '3g2z', '3g31', '3gbb', '3gc5', '3ge7', '3gnw', '3gr2', '3gv9', '3gy4', '3ivg', '3jvr', '3jvs', '3jya', '3k5v', '3kgp', '3kr8', '3kwa', '3l7b', '3lka', '3mss', '3myg', '3n76', '3n7a', '3n86', '3nq9', '3nw9', '3nx7', '3o9i', '3oe4', '3oe5', '3ozs', '3ozt', '3p5o', '3prs', '3pww', '3pxf', '3pyy', '3qgy', '3qqs', '3r88', '3rlr', '3rr4', '3rsx', '3ryj', '3syr', '3tsk', '3twp', '3u5j', '3u8k', '3u8n', '3u9q', '3udh', '3ueu', '3uev', '3uew', '3uex', '3ui7', '3uo4', '3up2', '3uri', '3utu', '3uuo', '3wtj', '3wz8', '3zdg', '3zso', '3zsx', '3zt2', '4abg', '4agn', '4agp', '4agq', '4bkt', '4cig', '4ciw', '4cr9', '4cra', '4crc', '4ddh', '4ddk', '4de1', '4de2', '4de3', '4djv', '4dld', '4dli', '4e5w', '4e6q', '4ea2', '4eky', '4eo8', '4eor', '4f09', '4f2w', '4f3c', '4f9w', '4gfm', '4gid', '4gkm', '4gr0', '4hge', '4ih5', '4ih7', '4ivb', '4ivc', '4ivd', '4j21', '4j28', '4j3l', '4jfs', '4jia', '4jsz', '4jxs', '4k18', '4k77', '4kz6', '4kzq', '4kzu', '4llx', '4lzs', '4m0y', '4m0z', '4mgd', '4mme', '4ogj', '4owm', '4pcs', '4qac', '4qd6', '4rfm', '4tmn', '4twp', '4ty7', '4u4s', '4w9c', '4w9h', '4w9i', '4w9l', '4wiv', '4x6p', '5a7b', '5aba', '5c28', '5c2h', '5dwr', '5tmn' |
| PDBbind v2020 | '6qqw', '6d08', '6jap', '6np2', '6uvp', '6oxq', '6jsn', '6hzb', '6qrc', '6oio', '6jag', '6moa', '6hld', '6i9a', '6e4c', '6g24', '6jb4', '6s55', '6seo', '6dyz', '5zk5', '6jid', '5ze6', '6qlu', '6a6k', '6qgf', '6e3z', '6te6', '6pka', '6g2o', '6jsf', '5zxk', '6qxd', '6n97', '6jt3', '6qtr', '6oy1', '6n96', '6qzh', '6qqz', '6qmt', '6ibx', '6hmt', '5zk7', '6k3l', '6cjs', '6n9l', '6ibz', '6ott', '6gge', '6hot', '6e3p', '6md6', '6hlb', '6fe5', '6uwp', '6npp', '6g2f', '6mo7', '6bqd', '6nsv', '6i76', '6n53', '6g2c', '6eeb', '6n0m', '6uvy', '6ovz', '6olx', '6v5l', '6hhg', '5zcu', '6dz2', '6mjq', '6efk', '6s9w', '6gdy', '6kqi', '6ueg', '6oxt', '6oy0', '6qr7', '6i41', '6cyg', '6qmr', '6g27', '6ggb', '6g3c', '6n4e', '6fcj', '6quv', '6iql', '6i74', '6qr4', '6rnu', '6jib', '6izq', '6qw8', '6qto', '6qrd', '6hza', '6e5s', '6dz3', '6e6w', '6cyh', '5zlf', '6om4', '6gga', '6pgp', '6qqv', '6qtq', '6gj6', '6os5', '6s07', '6i77', '6hhj', '6ahs', '6oxx', '6mjj', '6hor', '6jb0', '6i68', '6pz4', '6mhb', '6uim', '6jsg', '6i78', '6oxy', '6gbw', '6mo0', '6ggf', '6qge', '6cjr', '6oxp', '6d07', '6i63', '6ten', '6uii', '6qlr', '6sen', '6oxv', '6g2b', '5zr3', '6kjf', '6qr9', '6g9f', '6e6v', '5zk9', '6pnn', '6nri', '6uwv', '6ooz', '6npi', '6oip', '6miv', '6s57', '6p8x', '6hoq', '6qts', '6ggd', '6pnm', '6oy2', '6oi8', '6mhd', '6agt', '6i5p', '6hhr', '6p8z', '6c85', '6g5u', '6j06', '6qsz', '6jbb', '6hhp', '6np5', '6nlj', '6qlp', '6n94', '6.00E+13', '6qls', '6uil', '6st3', '6n92', '6s56', '6hzd', '6uhv', '6k05', '6q36', '6ic0', '6hhi', '6e3m', '6qtx', '6jse', '5zjy', '6o3y', '6rpg', '6rr0', '6gzy', '6qlt', '6ufo', '6o0h', '6o3x', '5zjz', '6i8t', '6ooy', '6oiq', '6od6', '6nrh', '6qra', '6hhh', '6m7h', '6ufn', '6qr0', '6o5u', '6h14', '6jwa', '6ny0', '6-Jan', '6ftf', '6oxw', '6jon', '6cf7', '6rtn', '6jsz', '6o9c', '6mo8', '6qln', '6qqu', '6i66', '6mja', '6gwe', '6d3z', '6oxr', '6r4k', '6hle', '6h9v', '6hou', '6nv9', '6py0', '6qlq', '6nv7', '6n4b', '6jaq', '6i8m', '6dz0', '6oxs', '6k2n', '6cjj', '6ffg', '6a73', '6qqt', '6a1c', '6oxu', '6qre', '6qtw', '6np4', '6hv2', '6n55', '6e3o', '6kjd', '6sfc', '6qi7', '6hzc', '6k04', '6op0', '6q38', '6n8x', '6np3', '6uvv', '6pgo', '6jbe', '6i75', '6qqq', '6i62', '6j9y', '6g29', '6h7d', '6mo9', '6jao', '6jmf', '6hmy', '6qfe', '5zml', '6i65', '6e7m', '6i61', '6rz6', '6qtm', '6qlo', '6oie', '6miy', '6nrf', '6gj5', '6jad', '6mj4', '6h12', '6d3y', '6qr2', '6qxa', '6o9b', '6ckl', '6oir', '6d40', '6e6j', '6i7a', '6g25', '6oin', '6jam', '6oxz', '6hop', '6rot', '6uhu', '6mji', '6nrj', '6nt2', '6op9', '6pno', '6e4v', '6k1s', '6a87', '6oim', '6cjp', '6pyb', '6h13', '6qrf', '6mhc', '6j9w', '6nrg', '6fff', '6n93', '6jut', '6g2e', '6nd3', '6os6', '6dql', '6inz', '6i67', '6quw', '6qwi', '6npm', '6i64', '6e3n', '6qrg', '6nxz', '6iby', '6gj7', '6qr3', '6qr1', '6s9x', '6q4q', '6hbn', '6nw3', '6tel', '6p8y', '6d5w', '6t6a', '6o5g', '6r7d', '6pya', '6ffe', '6d3x', '6gj8', '6mo2' |

The source of the PDBbind dataset can be found in http://www.pdbbind.org.cn (Only need a registration to download the data). The training set and validation set are all the other samples in the PDBbindv2016 and PDBbindv2020 after removing the test set mentioned above. The source code for training and data to train the model is available at https://github.com/gojx1998/LGN.
